# Weak acid-initiated slow release of Dexamethasone from hydrogel to treat orbital inflammation

**DOI:** 10.7150/thno.85627

**Published:** 2023-07-09

**Authors:** Jinjing Li, Aichi Zhang, Andi Zhao, Zhaoxia Chen, Gaolin Liang, Hu Liu, Chengfan Wu

**Affiliations:** 1 Department of Ophthalmology, The First Affiliated Hospital with Nanjing Medical University, Nanjing 210029, China.; 2 College of Biotechnology and Bioengineering, Zhejiang University of Technology, Hangzhou 310014, China.; 3 State Key Laboratory of Bioelectronics, School of Biological Science and Medical Engineering, Southeast University, Nanjing 210096, China.

**Keywords:** Orbital inflammation therapy, Supramolecular hydrogel, Sustained release, Dexamethasone

## Abstract

**Rationale:** Orbital inflammation is a prevalent and prolonged ocular disease that poses a significant challenge to clinicians. Glucocorticoid Dexamethasone sodium phosphate (Dex) has demonstrated efficacy in the clinical treatment of nonspecific orbital inflammation. However, frequent administration is required due to the short half-life of Dex, which may lead to drug waste and adverse side effects.

**Methods:** In this study, we co-assembled Dex with a weak acid responsive hydrogelator Py-Phe-Phe-Lys-Lys-OH (K) to obtain a novel supramolecular hydrogel Dex/K that could release Dex in a slow manner to treat orbital inflammation. The therapeutic effect of Gel Dex/K on orbital inflammation was verified by *in vitro* and *in vivo* experiments.

**Results:** *In vitro* experiments indicated that co-assembly of Dex with K significantly increased mechanic strength of the hydrogel, enabling a continuous release of 40% of total Dex within 7 days. *In vivo* experiments further demonstrated that sustained release of Dex from Gel Dex/K could effectively alleviate the infiltration of inflammatory cells and the release of inflammatory factors in the orbit of mice, improving symptoms such as increased intraocular pressure and proptosis. Additionally, Gel Dex/K mitigated the degree of tissue fibrosis and fatty infiltration by reducing the development of local inflammation in the orbit.

**Conclusions:** Our research results indicate that Gel Dex/K could more efficiently achieve responsive drug release in orbit, providing an innovative method for treating orbital inflammation.

## Introduction

Orbital inflammation is a common ophthalmic clinical disease, accouting for more than half of all orbital diseases. The main types of orbital inflammation are thyroid eye disease and nonspecific orbital inflammation 1, which includes a group of noninfectious lesions lacking characteristic pathological changes. These lesions, known as idiopathic orbital inflammation, are immune inflammations that occur in the tissues of the orbit 2. Nonspecific orbital inflammation primarily affects both eyes and is more common in adults, with no gender or ethnic differences observed 3. Nonspecific orbital inflammation affects various orbital tissues, including the retrobulbar fat, extraocular muscles, optic nerve, and surrounding adnexal structures 4, causing a variety of clinical manifestations, such as diplopia, proptosis, periorbital destruction, and compressive optic neuropathy, which ultimately leads to vision loss 5.

Systemic corticosteroids are the primary drug therapy for nonspecific orbital inflammation, with the first hormone therapy involving a sufficient amount and a full course of treatment to reduce the possibility of postoperative recurrence 6. Dexamethasone (Dex) is a kind of glucocorticoid with relative lipophilicity, which could easily penetrate biological membranes and is widely used in the clinical treatment of nonspecific orbital inflammation 7, 8. Studies have shown that Dex may inhibit the proliferation of orbital fibroblasts by downregulating the expression of intracellular ICAM-1, thus playing an anti-inflammatory and anti-fibrosis role 9, 10. However, long-term application of systemic drug therapy may lead to various side effects in patients, including hyperglycemia, diabetes, gastric ulcer, and posterior subcapsular cataract, etc 11. In addition, due to the short half-life of Dex routinely administered and the difficulty of maintaining an effective concentration in the lesion, ophthalmic treatment requires frequent administration (3-5 times per week for at least 1-2 months), which may lead to drug waste and adverse side effects, reduce patient compliance or increase the risk of infection 12, 13.

In view of the above situation, researchers proposed that nanomaterials based on micelles, liposomes or supramolecular hydrogels could provide a new alternative to improve the stability of therapeutic drugs and maintain the long-term retention in lesions 14-16. Among them, supramolecular hydrogels formed by the self-assembling of the hydrogelators into three-dimensional fibrous networks to gel large amount of water, have higher biosafety, higher drug loading efficiency and are easier to be metabolized 17-20. In recent years, they have been widely studied and applied in the biomedical field 21, 22. As a drug delivery system, supramolecular hydrogels are suitable for local injection and can prolong the residence time of drugs in the eye, thereby reducing drug waste and side effects 23, 24. Constructing environmentally sensitive hydrogels with stimuli-sensitive groups could achieve a sustained release of drugs in specific biological microenvironment 25. As a drug delivery system, it has been widely used in various medical applications and attracted the attention of researchers 13, 26, 27. Among them, pH-responsive hydrogel could control the amount and speed of drug release according to the pH changes caused by pathological conditions in spatial and temporal control 28, 29. However, to the best of our knowledge, there has been no report on the use of pH-responsive hydrogel to treat nonspecific orbital inflammation so far.

**Figure 1:**
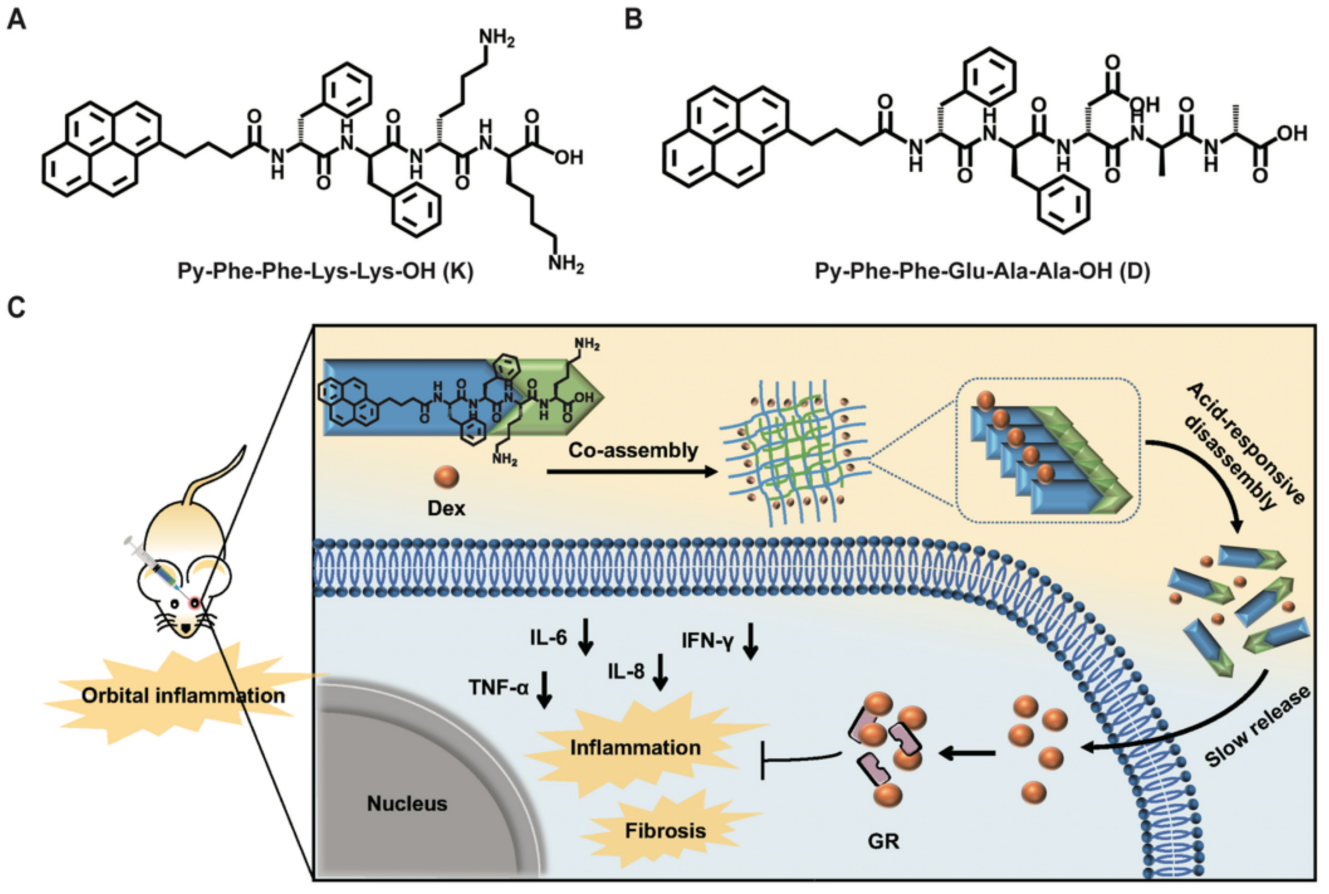
** Chemical structures of hydrogels and experimental design schematic. (A)** Chemical structures of Py-Phe-Phe-Lys-Lys-OH (K) and Py-Phe-Phe-Glu-Ala-Ala-OH (D). **(B)** Schematic illustration of the improvement of nonspecific orbital inflammation in mice through sustained release of Dex by orbital injection of Gel Dex/K.

Inspired by above pioneering studies, naturally we think, could we co-assemble Dex with a pH responsive supramolecular hydrogelator to construct a novel supramolecular hydrogel for slowly releasing Dex according to the pH changes of local inflammation, thus prolonging the retention time of Dex in the inflammation lesion, reducing the injection frequency and treating nonspecific orbital inflammation more effectively? To achieve this goal, we co-assembled Dex with a weak acid-sensitive hydrogelator Py-Phe-Phe-Lys-Lys-OH (K) to obtain a new supramolecular hydrogel Gel Dex/K. The rationales lie in: (1) Py-Phe-Phe is an effective unit of self-assembly to form supramolecular hydrogels. (2) Amino acid Lys in K is protonable which enables disassembly of hydrogel Dex/K in weak acid environment. (3) According to our previous literatures, Dex contains benzene ring structure and could be co-assembled with Py-Phe-Phe through weak interactions such as π-π stacking and hydrogen bonding 30. As far as we all know, the design of Gel Dex/K is completely new. Although the application of hydrogels on corneal and rhegmatogenous retinal detachment has been reported 31-33, there is little literature on using this kind of pH-responsive hydrogel to treat nonspecific orbital inflammation. For parallel studies, we also co-assembled Dex with a pH-insensitive hydrogelator Py-Phe-Phe-Glu-Ala-Ala-OH (D) to form Gel Dex/D (**Figure 1A-B**). *In vitro* experiments indicated that co-assembly of Dex with K significantly increased mechanic strength of the hydrogel, enabling a continuous release of 40% of total Dex within 7 days. *In vivo* experiments confirmed that Gel Dex/K could release Dex slowly and continuously after 7 days of injection into the orbit of mice, effectively alleviating local inflammatory reaction, reducing cell apoptosis and release of inflammatory cytokines (**Figure 1c**).

## Materials and Methods

**Preparations of Gel K, Gel Dex/K, Gel D and Gel Dex/D.** All these four hydrogels were prepared at room temperature (about 25 °C). Gel K was prepared according to the following procedures. Firstly, 2 mg solid powder of Py-Phe-Phe-Lys-Lys-OH (K) was dispersed in 200 µL phosphate buffer (PB, 20 mM, pH 7.4). Next, it was adjusted to dissolve gradually by 1 M hydrochloric acid (HCl) with the help of sonication and vortex. Then 1 M sodium hydroxide (NaOH) was added drop by drop and finally the formation of transparent and clear hydrogel was observed. For preparation of Gel Dex/K, 2 mg K and 0.5 mg Dex (4: 1 mass ratio) were evenly dispersed in 200 µL PB, then Gel Dex/K (Final Dex concentration: 2.5 mg/mL) was prepared following the preparation procedures of Gel K. Gel D was prepared according to the similar procedures. Firstly, 2 mg solid powder of Py-Phe-Phe-Glu-Ala-Ala-OH (D) was dispersed in 200 µL PB. Next, it was adjusted to dissolve gradually by 1 M NaOH with the help of sonication and vortex. Then 1 M HCl was added drop by drop and finally the formation of transparent and clear hydrogel was observed. For preparation of Gel Dex/D, 2 mg D and 0.5 mg Dex (4: 1 mass ratio) were evenly dispersed in 200 µL PB, then Gel Dex/D (Final Dex concentration: 2.5 mg/mL) was prepared following the preparation procedures of Gel D. Stirring was not required in the whole process. All prepared hydrogel samples were stored at 4 °C**.**

**Cumulative release of Dex from Gel Dex/K or Gel Dex/D *in vitro*.** Firstly, using HPLC analysis, we obtained the standard calibration curves of Dex by plotting the relationship between Dex concentrations and its HPLC peak areas. PBS at different pH was bought from Shanghai Baoman Biotechnology Co., LTD. Take the cumulative release under pH 7.4 as an example. After preparing Gel Dex/K or Gel Dex/D as described above, we added 600 µL PBS (0.01 M, pH 7.4) to 500 µL Gel Dex/K or Gel Dex/D respectively, and incubated it at 37 °C. At different times (1, 2, 3, 5, 7 day), 100 µL of supernatant was collected for HPLC analysis and it was repeated three times in parallel. Then the culture mixture was replenished with the same volume of PBS taken as release medium immediately.

**Isolation, culturing and validation of primary orbital fibroblast cells from mice orbits.** We isolated orbital and surrounding tissue fragments from the eyes of experimental animal donors. In a sterile environment, after removing adipose tissue, we washed them three times in pre-cooled PBS containing dual antibodies and decomposed the connective tissue into fragments. After moistening the cell culture plate with complete culture medium, we carefully attached the tissue to the cell culture plate. Then we inverted the cell culture plate with tissue attached in the incubator to allow the tissue to adhere to the wall as soon as possible. After 30 min, we added the complete culture medium to the cell culture plate and incubated cells for several days. After 3 days, we replaced half of the culture medium, observed cell growth the next day, and added appropriate culture medium. About 8-10 days later, cells will crawl out. The extracted cells were identified as orbital fibroblasts by immunofluorescence staining with anti-Vimentin antibody.

***In vitro* cytotoxicity assay.** Orbital fibroblasts cells (OF cells) were planted in 96-well plate (3 × 10^3^ cells per well) at an atmosphere of 37 °C and 5% CO_2_ overnight. Then the medium was replaced with IL-1β containing medium (10 ng/mL) to simulate the inflammatory environment* in vitro*. After determining the Dex concentration, to further evaluate the cytotoxicity of the hydrogels, we used cell counting kit-8 (CCK-8) to study the survival and proliferation of mice OF cells. We seeded 100 µL culture medium into a well of a cell culture plate as a control group (Ctrl). 20 µL of Gel D at 10 mg/mL in 100 µL culture medium, 20 µL of Gel Dex/D at 10 mg/mL in 100 µL culture medium, 20 µL of Gel K at 10 mg/mL in 100 µL culture medium, and 20 µL of Gel Dex/K at 10 mg/mL in 100 µL culture medium were seeded as experimental groups into the wells of a cell culture plate. Then each of above wells was added with 100 µL cell dispersion (3000 cells) in culture medium. The cell plates were incubated at 37 °C for 72 h for cytotoxicity study. After removing the medium, the sample were determined at 570 nm using a microplate reader.

**Idiopathic orbital inflammatory pseudotumor animal model.** Female BALB/c mice (8 weeks old) were purchased from Vital River Laboratories (Beijing, China). All experimental mice were housed and operated in an SPF-grade sterile environment. All experimental reagents were sterilized by UV light before being used. The mice were sensitized by a topical application of 2% oxazolone (4-ethoxymethylene-2-phenyl-2-oxazolone-5-one; E0753, Sigma-Aldrich, USA) solution in olive oil/acetone (2:1 vol/vol) to the shaved abdomen. Five days later, we injected 5 µl of 2% oxazolone solution into the right orbit of the experimental group of mice through the posterior of the sub-Tenon's using a 30-gauge needle, while the left orbit was injected with Phosphate Buffered Saline (PBS) as a sham group. Then we randomly divided the successfully molded mice into five groups (n=5) and injected 10 μL of Dexamethasone sodium phosphate solution (2.5 μg/μL) or Dexamethasone sodium phosphate hydrogel (12.5 μg/μL) into the right orbit of the mice, respectively.

**Pathological staining.** Experimental animals were euthanized after oxazolone injection or Dexamethasone treatment for 72 h, 5 days, and one week. Tissue from orbital exenteration was fixed in 4% paraformaldehyde (PFA; 15714-5, Electron Microscopy Sciences) to perform histopathologic and immunohistochemical analysis. On day 7, major tissues of mice were fixed with 10% formalin over 48 h, embedded in paraffin, cut into 5 μm sections for pathological staining, and evaluated using light microscopy.

**Immunohistochemistry staining.** At the end of therapy, the mice were sacrificed by overdose anesthesia and orbits tissues were exenterated and fixed in 4% paraformaldehyde for analysis. Centering the specimen on the optic nerve, the orbital tissue was oriented for sagittal paraffin sections. Immunofluorescence staining sections were deparaffinized in 100% xylene, rehydrated, and rinsed with PBS. After heat-induced epitope retrieval and washing with PBS, sections were blocked for one hour at room temperature using blocking buffer composed of 0.01% Triton X-100 (T8787-100ML; Sigma-Aldrich). The primary antibodies, CD3 (ab16669, 1:100; abcam), Ly6G (ab25377, 1:100; abcam), IBA1 (ab5076, 1:100; abcam) were incubated overnight at 4 °C. The following day, sections were incubated for four h in secondary antibodies CoraLite 488-conjugated Goat Anti-Rabbit IgG (H+L) (SA00013-2, 1:100; Proteintech). DAPI was used for nuclear staining and counting.

**Flow cytometry.** We used the membrane linked protein V-FITC apoptosis detection kit (Beyotime) to detect OF cells after different treatments. Cells were isolated from culture flasks by trypsin, centrifuged (1000 g) and collected. We took 50,000 - 100,000 cells and resuspended the cells with 200 μL of membrane linked protein V-FITC adduct and 10 μL of propidium iodide staining solution and mixed them gently. Cells were incubated at room temperature and protected from light for 10 - 20 min before detection using flow cytometry (Beckman). After extracting mice orbital tissue, we fixed the tissue and collected the cells. T cells and macrophages were specifically labeled using the antibodies described above and dark stained at room temperature for 30 min prior to performing machine analysis. We record fluorescent event counts and analyze the data using CytExpert software.

**Statistical analysis.** Data analysis was performed using GraphPad Prism 8 (GraphPad Software, San Diego, CA). All the data were presented as means ± SD. For the normally distributed data with equal variance, the significant difference was determined by Student's t-test (when two groups were compared) or one-way or two-way ANOVA to test the effect of group (when > 2 groups were compared). For the non-normally distributed data or data with unequal variances, the significant difference was determined by non-parametric Mann-Whitney's U-test (when two groups were compared) or Kruskal-Wallis's test followed by post-hoc Bonferroni's test (when > 2 groups were compared). P < 0.05 was considered statistically significant. All experiments were replicated at least three times.

## Results and Discussion

### Preparations of Gel K, Gel D, Gel Dex/K and Gel Dex/D

**Figure 2:**
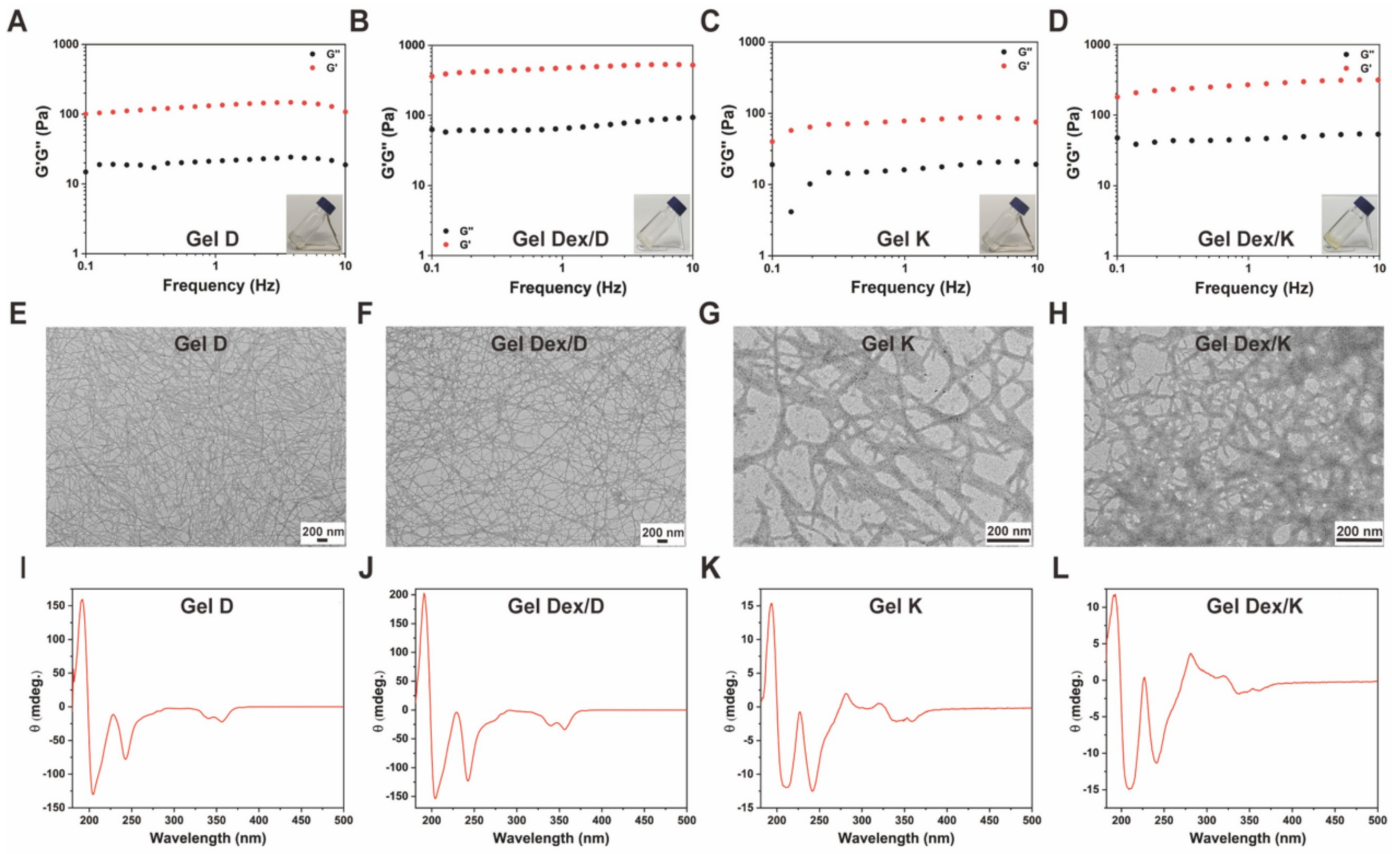
** Characterization of hydrogels. (A-D)** The dynamic storage moduli (G') (red) and the loss moduli (G'') (black) of 10 mg/mL Gel D, Gel Dex/D, Gel K and Gel Dex/K (insets: photographs of Gel D, Gel Dex/D, Gel K and Gel Dex/K). **(E-H)** TEM images of 10 mg/mL Gel D, Gel Dex/D, Gel K and Gel Dex/K, respectively. Scale bar: 200 nm. **(I-L)** CD spectra of 10 mg/mL Gel D, Gel Dex/D, Gel K and Gel Dex/K, respectively.

After syntheses and characterizations of Py-Phe-Phe-Lys-Lys-OH (K) and Py-Phe-Phe-Glu-Ala-Ala-OH (D) (**Schemes S1-S2, Figure S1-S6**), we investigated their hydrogelation conditions with or without Dex according to previous literature and the detailed procedure was described in the supporting information 34. As can be seen from the inset images in** Figure 2A-D**, the hydrogels loaded with (Gel Dex/K and Gel Dex/D) or without Dex (Gel K and Gel D) are both transparent and clear, which indicated that Dex co-assembles but not physically mix with Py-Phe-Phe-Lys-Lys-OH (K) or Py-Phe-Phe-Glu-Ala-Ala-OH (D) to form the corresponding hydrogel (**Tables S1-S2**).

### CACs of Gel D, Gel Dex/D, Gel K and Gel Dex/K

To further determine the gelation ability of the four hydrogels (Gel K, Gel D, Gel Dex/K and Gel Dex/D), we investigated their critical aggregation concentration (CACs). The results showed that the critical aggregation concentration of Gel D, Gel Dex/D, Gel K and Gel Dex/K were 15.48, 15.84, 16.20 and 34.06 μM respectively (**Figure S7**). The similar CAC values of these four hydrogels indicated that they had similar self-assembly capabilities.

### Rheology, TEM characterizations, and CD spectra of Gel D, Gel Dex/D, Gel K and Gel Dex/K

Next we used rheology to investigate the viscoelastic properties of Gel D, Gel Dex/D, Gel K and Gel Dex/K. As shown in **Figure 2A-D** and **Figure S8**, the storage modulus (G') of the four hydrogels were significantly higher than their loss modulus (G'') in the investigated frequency range (0.1 - 10% Hz) and strain range (0.1 - 10%), respectively, indicating all these four hydrogels could tolerate external forces. Meanwhile, the G' and G'' values of the hydrogels loaded with Dex were higher than those of hydrogels without Dex, indicating that the former had higher mechanical strength. Then we conducted transmission electron microscopy (TEM) observation to investigate the internal networks in these four hydrogels. As shown in **Figure 2E-H**, all the hydrogels were composed of long and flexible nanofibers. After TEM observation, we used circular dichroism (CD) spectra to study the molecular packing of these hydrogels (Gel K, Gel D, Gel Dex/K and Gel Dex/D) (**Figure 2I-L**). Similar CD spectra of the four hydrogels indicated that they have similar secondary structures. In particular, a remarkable Cotton effect at around 230 nm and a clear negative CD absorption at around 240 nm in CD spectrum were observed and they indicated the formation of β-sheet secondary structures in these four hydrogels 35. All these above results suggested that the encapsulation of Dex almost did not interfere with the molecular arrangements and microscopic morphology in these hydrogels, but obviously enhanced the mechanical strength of the related hydrogels, which made them suitable for injection therapy in the orbital region.

### Cumulative release of Dex from Gel Dex/K or Gel Dex/D *in vitro*

**Figure 3:**
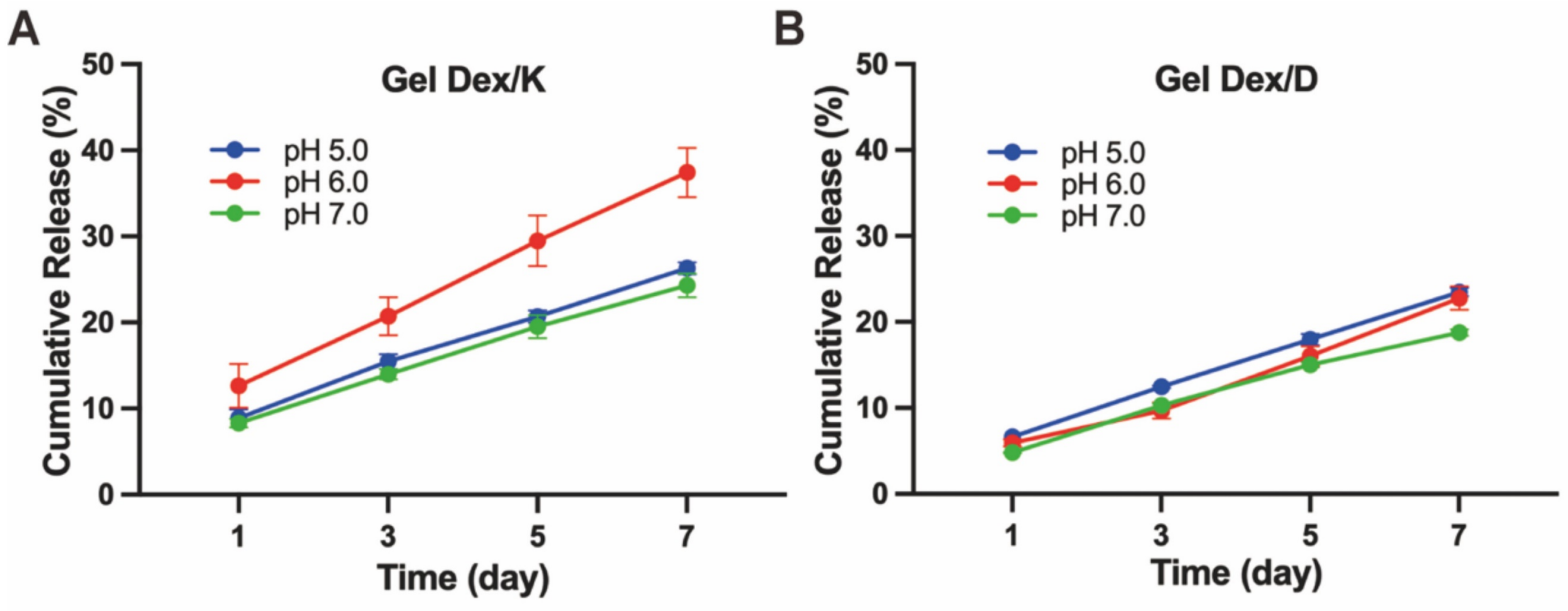
**Hydrogel releases drugs slowly *in vitro*.** *In vitro* cumulative releases of Dex from Gel Dex/K **(A)** or Gel Dex/D **(B)** in PBS (0.01 M, pH 5.0 - 7.0) at 37 °C, respectively.

We next investigated the release behaviour of Gel Dex/K and Gel Dex/D *in vitro*, respectively (**Figure S9, Table S3**). As shown in **Figure 3**, continuous release of Dex within one week was observed under different pH values (5.0 - 7.0) mimicking orbital inflammation environment *in vitro* 36. The results of *in vitro* release monitoring showed that Dex was released from Gel Dex/K in a sustainable manner under mildly acidic conditions, and the cumulative release increased with time. The sustained-release effect was the best under pH 6.0, and the drug release amount on the seventh day could reach 37.4 ± 2.9% of the total. Under the condition of pH 5.0, the release rate of Dex from Gel Dex/K is slower than that under the condition of pH 6.0. What's more, since Gel Dex/D was pH-insensitive, the drug release amount on the seventh day is 22.8 ± 1.4% of the total amount under pH 6.0, and the sustained release effect is inferior to that of Gel Dex/K. All these above experimental results indicated that Gel Dex/K could release Dex in a sustainable manner in a weak acid environment and is more suitable for the treatment of orbital inflammation.

### Cytotoxicity evaluation of Dex, Gel D, Gel Dex/D, Gel K and Gel Dex/K

After *in vitro* characterizations, in order to explore the suitable concentration range of Dex in our experiments, we seeded cells into 96-well plates and treated them with different concentrations of Dex for 24 h.

**Figure 4:**
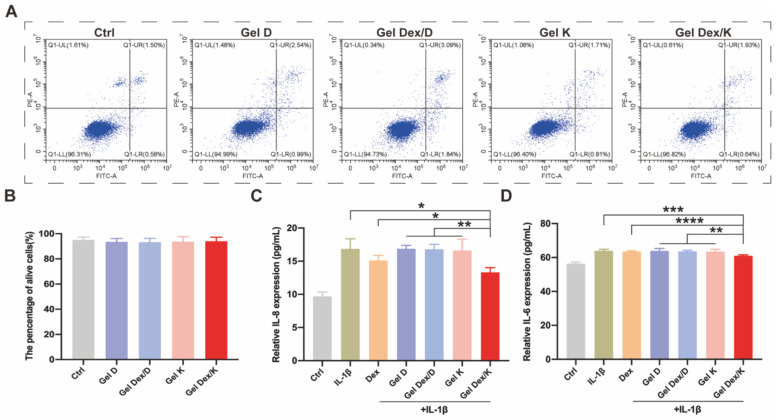
**Expression levels of apoptosis and inflammatory factors *in vitro*. (A)** Apoptosis detection of OF cells co-incubated with each group of hydrogels. **(B)** The proportion of surviving cells in each group after co-incubated with the hydrogels. **(C-D)** Quantitative analysis of the expression levels of inflammatory factors in OF cells in a simulated inflammatory environment *in vitro*; n = 5 for each group. *P < 0.05, **P < 0.01, ***P < 0.001, ****P < 0.0001.

The results showed that Dex was non-cytotoxic to orbital fibroblasts cells (OF cells) at the concentrations ranging from 0.01 to 1 mg/mL (**Figure S10**), so we chose 0.25 mg/mL for following experiments. As shown in **Figure S11**, after co-incubated with the four hydrogels, the cell viability and survival status of each group were not significantly different from the Ctrl group, suggesting that the hydrogels co-assembled with (or w/o) Dex were highly compatible with OF cells. Furthermore, a flow cytometric apoptosis experiment by PI/Annexin V kit was carried out to verify the effect of these four hydrogels on apoptosis of OF cells. Consistent with the above experimental steps, we inoculated 20 μL of each hydrogel into the wells of the cell culture plate, and co-cultured them with OF cells for 72 h. By analyzing the proportion of apoptotic cells and viable cells, we found that there was no statistical difference in the proportion of viable cells among the groups (**Figure 4A-B**). Collectively, these results indicated that none of these four hydrogels affected the viability of OF cells and could be applied for following *in vivo* experiments.

### Apoptosis and inflammatory factor expression analyses in inflammatory environment *in vitro*

Next, we investigated the effect of hydrogels on cell apoptosis and inflammatory factor expression in a simulated inflammatory environment *in vitro*. 10 ng/mL of IL-1β was added to the cell culture medium as a stimulating factor to simulate the inflammatory environment* in vitro* 37. After 24 h of stimulation, the cells were further cultured in medium containing 0.25 mg/mL Dex or 10 mg/mL hydrogels. 20 μL of different hydrogels were spread in the wells of the cell culture plate, and 200 μL of IL-1β containing medium was added to each well. The cells were then cultured at 37 °C for 72 h, and the ratio of viable cells and apoptotic cells was analyzed by flow cytometry. As shown in **Figure S12**, the apoptosis rate under the stimulation of inflammatory environment* in vitro* (5.2 ± 0.7) was obviously higher than that of the Ctrl group (1.6 ± 0.6). Compared with IL-1β stimulated group or other experimental groups, the percentage of apoptosis in Dex and Gel Dex/K group was decreased by 2.6 ± 1.3 and 2.3 ± 1.2, respectively. However, there was no significant difference between the two groups, which may be due to the cell culture time was not long enough, and the sustained-release effect of the drug was not obvious.

In addition to inhibition of apoptosis, Dex could also activate the transcription of target genes by binding to glucocorticoid receptors (GR), affecting the activation of multiple signaling pathways such as NF-κB, ultimately exerting anti-inflammatory and anti-immune effects 38.

IL-6 and IL-8 are considered to be important inflammatory factors in orbital inflammation 39. Following IL-1β stimulation and treatment of Dex or different hydrogels for 72 h, cell supernatants were collected for ELISA assays as described above for cells. As shown in **Figure 4C-D**, both Dex and Gel Dex/K significantly reduced the expression of inflammatory factors under IL-1β stimulation *in vitro*. Quantitative analysis indicated that the expression of IL-8 in the cell culture medium was 16.9 ± 1.5 pg/mL under IL-1β stimulation, 15.1 ± 0.8 pg/mL in the Dex alone treatment group and 13.3 ± 0.7 pg/mL in the Gel Dex/K group. The expression of IL-6 in cell culture medium was 64.0 ± 0.9 pg/mL in IL-1β-stimulated group, 63.6 ± 0.4 pg/mL in the Dex group and 61.0 ± 0.5 pg/mL in the Gel Dex/K treatment group. In general, although Dex could ameliorate the expression of inflammatory factors *in vitro*, its anti-inflammatory effect was further enhanced when co-assembled with Gel K. To further explore whether this anti-inflammation effect in Gel Dex/K was acid environment-dependent, we took Gel Dex/D as a negative control and observed whether it could achieve the effect of sustained drug release. The results verified that the expression of the above inflammatory factors in Gel Dex/D group did not decrease and previous inflammation-suppressive efficacy of Dex was abolished, which demonstrated that Gel Dex/D does not release Dex and exert anti-inflammatory effect in acidic environment.

Our results showed that both Dex and Gel Dex/K significantly reduced the expression of inflammatory factors under IL-1β stimulation *in vitro*, making them potential therapeutic options for the treatment of orbital inflammation.

### Analysis of intraocular pressure and inflammatory factor expression in mice with orbital inflammation

**Figure 5:**
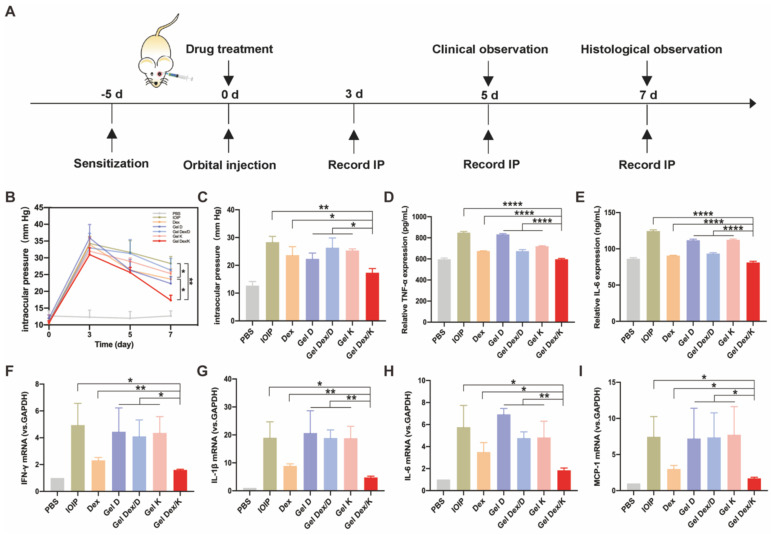
**Expression levels of intraocular pressure and inflammatory factors in 7 groups of mice. (A)** Treatment timeline for *in vivo* experiments. **(B)** IOP monitoring for 7 consecutive days. **(C)** Quantitative analysis of intraocular pressure on the seventh day after treatment. **(D-E)** Quantitative analysis of the expression levels of inflammatory factors (TNF-α; IL-6) in 7 groups of mice after orbital inflammation. **(F-I)** Quantitative analysis of mRNA expression of inflammatory factors (IFN-γ, IL-1β, IL-6 and MCP-1) after orbital inflammation in 7 groups of mice. n = 3 for each group. *P < 0.05, **P < 0.01, ***P < 0.001, ****P < 0.0001.

After* in vitro* characterization and evaluation of cytotoxic and anti-inflammatory effects of Gel D, Gel Dex/D, Gel K and Gel Dex/K, we investigated their therapeutic effects on orbital inflammation in mice. We adopted an idiopathic obital inflammatory pseudotumor (IOIP) model to evaluate the therapeutic efficacy of these four hydrogels 40. We selected 8-week-old BALB/c mice for following studies, and applied 2% oxazolone solution (4-ethoxymethylene-2-phenyl-2-oxazolone-5-one; Sigma) in olive oil/acetone (2:1 vol/vol) on the abdomen to sensitize the mice. Five days after sensitization, the orbit was challenged by a sub-Tenon's retrobulbar injection with 5 μL of 2% oxazolone solution using 30-gauge, 1/2-inch needle. The PBS group was the control group that was not injected oxazolone to induce inflammation, and only injected the same amount of PBS. Mice in other experimental groups were not injected with oxazolone in the left orbit, while injected in the right orbit. After inducing inflammation, each experimental group was injected with 10 μL of Dex (2.5 mg/mL) or 10 mg/mL each of hydrogel in the right orbit, respectively. Then we performed the following animal experiments according to a defined schedule (**Figure 5A**).

As we know, orbital inflammation could lead to clinical manifestations such as new lipogenesis, hyaluronic acid synthesis, interstitial edema, and increased intraocular pressure 41. Topical corticosteroids also carry a risk of transient intraocular pressure elevation 11. So we recorded intraocular pressure (IOP) from three mice in each group at three time points, 3 days, 5 days and 1 week after drug injection. Three days after orbital injection, the intraocular pressure of all experimental mice was significantly increased, compared with the control group without injection. With the passage of time, the intraocular pressure decreased slightly on day five. On the seventh day, we quantified the differences in IOP between these groups and found that Dex and Gel Dex/K had a suppressive effect on IOP elevation compared to the untreated group (IOIP) (**Figure 5B**).

Quantitative analysis showed that on the seventh day after Gel Dex/K treatment, the IOP value decreased from 31 ± 2 to 17 ± 2. Although the effects of free Dex and Gel Dex/K treatments on IOP were similar, by comparing the differences between the two groups, we believe that the latter modulates IOP more significantly. In addition, Gel D and Gel Dex/D had no significant effect on decreasing IOP compared to the untreated group (**Figure 5C**). These results suggested that Gel Dex/K had better effect on treating the elevated IOP caused by orbital inflammation, which might be due to the sustained release of the Dex in the hydrogel.

TLRs are major mode receptors that recognize a variety of microbial products and mediate inflammatory responses by producing various chemokines and cytokines involved in microbial clearance 42. Among the mediators involved in TLR transduction, IL-1β, MCP-1, IL-6 and IFN-γ play crucial roles 43. One of the main effects produced by these mediators is the recruitment and infiltration of various inflammatory response-related cells to the lesion site. These cytokines are rapidly released when orbital fibroblasts are activated by stimuli in an inflammatory environment 44. We therefore assessed the secretion and mRNA expression of the above cytokines in the IOIP model. The experimental grouping was the same as above, and the periorbital tissue homogenate was collected for ELISA and PCR detection. As shown in **Figure 5D-E**, treatment with free Dex and Gel Dex/K could significantly inhibit the level of TNF-α cytokines in inflammatory state, with the Gel Dex/K group decreasing to 597.5 ± 6.2; however, treatment with Gel Dex/D was not obvious. The expression of cytokine IL-6 showed the same trend, with cytokine expression in the Gel Dex/K group decreasing to 81.1 ± 1.5, meaning that Gel Dex/K treatment had the best inhibitory effect on inflammatory factors. These results were consistent with the findings of previous studies that Dex exert anti-inflammatory effects by blocking the expression of cytokines such as interleukins. Gel Dex/K could continuously release Dex in response to the local inflammatory environment, further enhancing the anti-inflammatory effect.

Then we used PCR technology to detect cytokines IL-1β, IL-6, MCP-1 and IFN-γ for further exploring their expression levels at the RNA level. The inflammatory response in the orbital site resulted in an obvious increase in the levels of the above cytokines. Dex and Gel Dex/K treatment showed strong anti-inflammatory activities by inhibiting the expression of these cytokines, and both showed similar effects (**Figure 5F-I**). Quantitative analysis showed that, compared with Gel K, Gel D and Gel Dex/D groups, both Dex and Gel Dex/K treatments could effectively inhibit the elevation of these inflammatory factors in IOIP, and Gel Dex/K treatment had the best inhibitory effect on these inflammatory factors. Compared with IOIP group, Gel K, Gel D and Gel Dex/D groups had no significant difference in the expression levels of inflammatory factors. We considered that Gel Dex/K could respond to changes in the inflammatory environment at the lesion, releasing the drug slowly and achieving a better anti-inflammatory effect. All these above results together suggested that: 1) Gel K, Gel D, and Gel Dex/D did not inhibit the elevation of inflammatory factors in IOIP; 2) Although Dex can improve the inflammatory response in IOIP, its anti-inflammatory effect is also enhanced when Dex is co-assembled with the hydrogelator K and then Dex is released in a sustainable manner.

### Effects on Orbital Histomorphology

The above results indicated that Gel Dex/K had better inhibitory effects on apoptosis and inflammation in IOIP mice than Dex and Gel Dex/D. As we know, the accumulation of inflammatory cells, tissue fibrosis and hyperplasia, and fat accumulation will occur during the development of inflammation 45.

**Figure 6:**
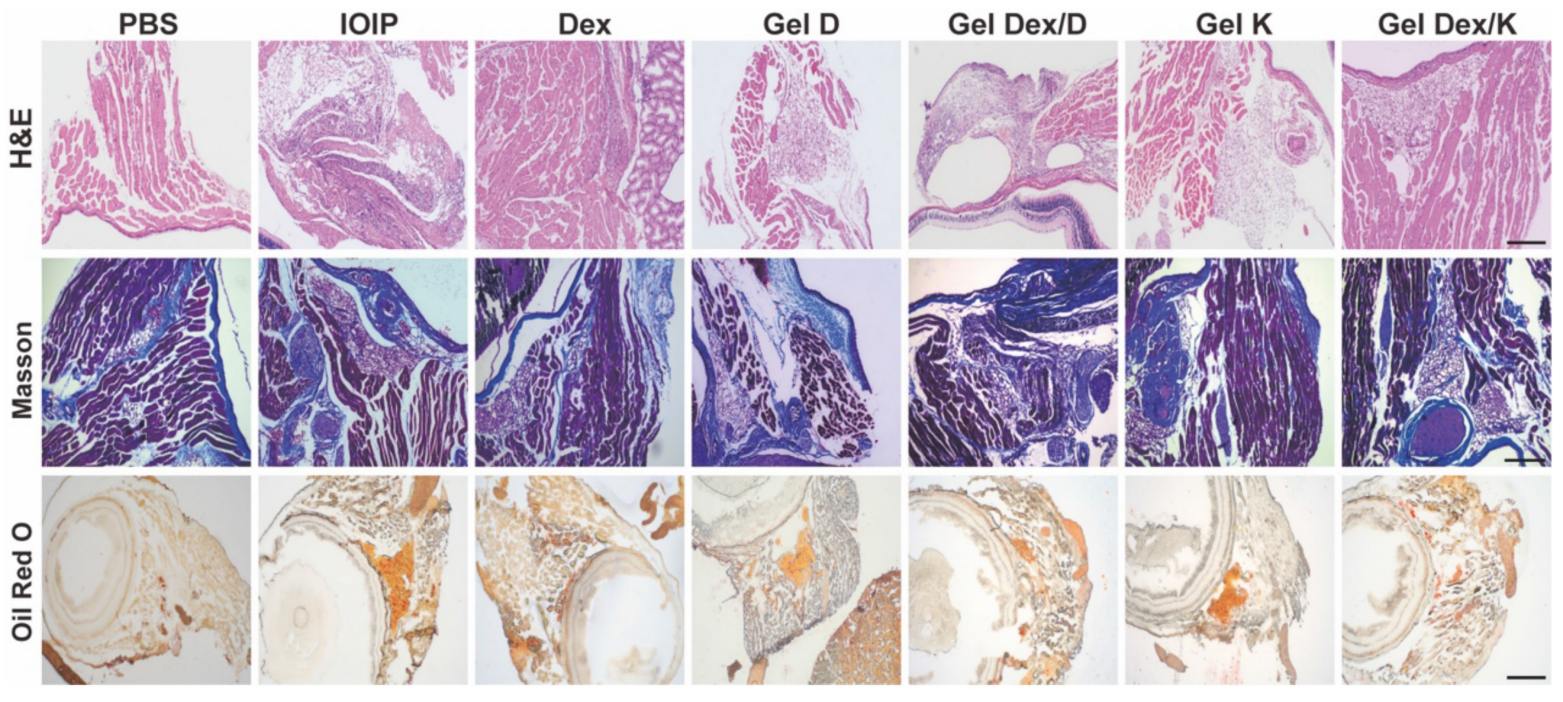
** Morphological changes in orbital inflammation were assessed using pathological section staining on the seventh day after treatment.** Representative H&E staining, Masson's staining, and Oil Red O staining images of mice orbital sections of 7 groups. Scale bar: 100 μm. n = 3 for each group.

The present study investigated the effects of Gel Dex/K, Dex, and Gel Dex/D on the orbital histomorphology in IOIP mice. The results showed that Gel Dex/K had better inhibitory effects on apoptosis and inflammation than Dex and Gel Dex/D. Inflammation can lead to tissue fibrosis, hyperplasia, and fat accumulation; therefore, we analyzed the morphological changes in the orbital tissue of IOIP mice in each treatment group. Histological observation showed that the hydrogel had a high biosafety and did not cause significant structural changes in the retina or other sites. Gel Dex/K treatment significantly reduced inflammatory infiltration and preserved normal retinal structures, as confirmed by H&E and Masson's staining. Gel Dex/K also inhibited the proliferation of collagen fibers and reduced fatty infiltration in the orbital tissue (**Figure 6**). The therapeutic effects of Gel K, Gel D, and Gel Dex/D were insignificant, as the latter failed to release Dex in response to local inflammation. Clinical findings showed that Gel Dex/K had a stronger protective effect against inflammation than the other drug-treated groups. Gel Dex/D did not respond to changes in the inflammatory environment and had no apparent therapeutic effect on orbital inflammation. These results suggest that Gel Dex/K exerts its acid-responsive sustained-release properties *in vivo* and relieves clinical symptoms without affecting the intrinsic morphological structure of the eyeball.

In order to visually observe the changes in the anterior segment of mice, we used a slit lamp to take photos of the ocular surface of mice on the fifth day after injection. All of the sensitized mice exhibited dermatitis and inflammation in the eyelids of the injected side and edema in the periorbital region as observed. After five days, proptosis of the injected eye with eyelid edema was still observed. The inflammation and proptosis of the mice in the Gel Dex/K group were decreased, but still present, exhibited a stronger protective effect against inflammation than the other groups (**Figure S13**). These clinical findings were contrast to the uninjected orbits of mice, which did not show any phenotypic changes. However, in terms of the Gel Dex/D treatment, both Gel D and Gel Dex/D failed to attenuate the inflammatory response, which was not significantly different to untreated group. These results suggested that the therapeutic effect of Gel Dex/K is superior to that of Gel Dex/D and free Dex. Gel Dex/D did not respond to changes in the inflammatory environment and released Dex, thus there was no apparent therapeutic effect on orbital inflammation. All the results indicated that Gel Dex/K exerted its acid-responsive sustained-release properties *in vivo* and relieved clinical symptoms.

From a morphological point of view, the above results suggested that orbital injection of hydrogel has no effect on the intrinsic morphological structure of the eyeball. Gel Dex/K treatment has a significant inhibitory effect on inflammatory cell infiltration, collagen fiber proliferation and adipose tissue deposition by continuously releasing Dex at the site of inflammation, and its effect is better than that of free Dex treatment.

### Effects on inflammatory cell infiltration in orbital tissue

**Figure 7:**
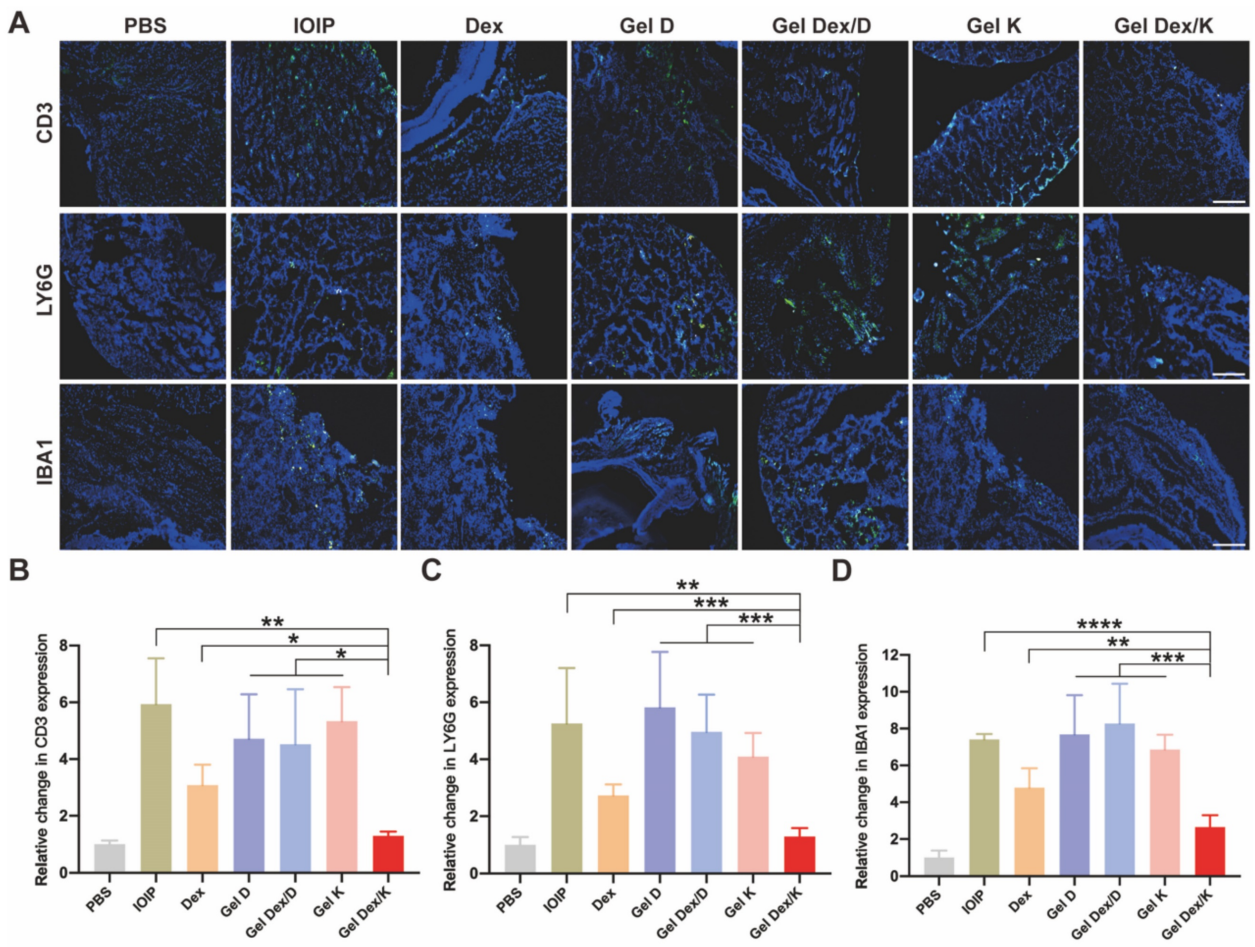
** Analysis of the infiltration of inflammatory cells in the orbits of mice in 7 groups on the seventh day after treatment. (A)** Representative images of infiltration of inflammatory cells (T cells, neutrophils, macrophages) in the orbits of mice in seven groups on the seventh day after treatment. **(B-D)** Immunofluorescence quantitative statistics of inflammatory cells in the orbital area of mice in each group. Scale bar is 100 μm. n = 3 for each group. *P < 0.05, **P < 0.01, ***P < 0.001, ****P < 0.0001.

The accumulation and infiltration of inflammatory cells in the orbit are key characteristics of the development of orbital inflammation. Dexamethasone is known to suppress immune inflammatory responses by inducing apoptosis of activated lymphocytes, particularly T cells. To gain a clearer understanding of the inflammatory response in the orbital tissue, we used immunofluorescence experiments to specifically label T cells (CD3), neutrophils (LY6G), and macrophages (IBA1) which play important roles in orbital inflammation. DAPI was used to co-stain all specimens to identify nuclei. Our examination of orbital tissue revealed a focal distribution of inflammatory cells in muscle and adipose tissue (**Figure 7A**). We observed a significant reduction in inflammatory cell infiltration after treatment with Gel Dex/K. Fluorescence quantitative statistics showed that compared with the IOIP mice, the infiltration of inflammatory cells was improved after Dex and Gel Dex/K treatment, while no significant difference was observed between the blank vehicle Gel K, Gel D, and Gel Dex/D treatment groups. The Gel Dex/K group showed the most significant treatment effect with the least count of inflammatory cells among all the treatment groups (**Figure 7B-D**). In order to obtain a more specific understanding of the expression of inflammatory cells in tissues, we used flow cytometry to detect the differences in the number of CD3^+^ T cells in the orbital tissues of mice in each group (**Figure S14**). The test results were consistent with the trend of the above immunofluorescence experiments. These results suggest that Gel Dex/K could effectively reduce the accumulation and infiltration of inflammatory cells in the orbital tissue, thereby improving the occurrence and development of orbital inflammation.

## Conclusions

In conclusion, in order to more effectively treat orbital inflammation, reduce the risk of infection and other side effects, we co-assembled Dex with a weak-acid responsive hydrogelator Py-Phe-Phe-Lys-Lys-OH (K) to prepare a novel supramolecular hydrogel Dex/K for sustained release of Dex. Rheology and TEM tests of Gel Dex/K showed that the co-assembly of Dex with K increased mechanical strength of Gel K, enabling Gel Dex/K more suitable for orbital injection to treat nonspecific orbital inflammation. Cumulative release profile of Dex from Gel Dex/K *in vitro* indicated that Dex was released from Gel Dex/K at a sustainable manner within 7 days. Cell experiments indicated that Gel Dex/K had no significant effect on the cell viability or apoptosis rate of OF cells. In a simulated inflammatory environment, Gel Dex/K released Dex more effectively and inhibited the expression of inflammatory factors. *In vivo* experiments indicated that Gel Dex/K sustained-release Dex could effectively relieve the infiltration of inflammatory cells and the release of inflammatory factors in the orbit of mice, improving symptoms such as increased intraocular pressure and proptosis. Furthermore, Gel Dex/K reduced the degree of tissue fibrosis and fatty infiltration by alleviating the development of local inflammation in the orbit. These results supported that Gel Dex/K could be served as a new ophthalmic drug delivery system to improve inflammatory local fibrosis, fat deposition and thus treat non-specific orbital inflammation. We anticipate that our Gel Dex/K could be used to treat non-specific orbital inflammation as well as other eye diseases in clinic in the near future.

## Supplementary Material

Supplementary methods, figures and tables.
