# Supplementary material for: Weak acid-initiated slow release of Dexamethasone from hydrogel to treat orbital inflammation
Source: Theranostics. 2023 Jul 9;13(12):4030–41. doi: 10.7150/thno.85627 (PMC10405854; doi:10.7150/thno.85627)
Supplement: Supplementary file 1 — Supplementary methods, figures and tables. [file thnov13p4030s1.pdf]

Supplementary Material

Weak acid-initiated slow release of Dexamethasone from hydrogel to treat orbital inflammation

Jinjing Li,[1] Aichi Zhang,[2] Andi Zhao,[1] Zhaoxia Chen,[3] Gaolin Liang,[*3] Hu Liu,[*1] and Chengfan Wu[*2]

1. Department of Ophthalmology, The First Affiliated Hospital with Nanjing Medical University, Nanjing 210029, China
2. College of Biotechnology and Bioengineering, Zhejiang University of Technology, Hangzhou 310014, China
3. State Key Laboratory of Bioelectronics, School of Biological Science and Medical Engineering, Southeast University, Nanjing 210096, China

E-mail: wucf0220@zjut.edu.cn. (C.-F. W.), liuhu@njmu.edu.cn. (H. L.), gliang@seu.edu.cn (G.-L. L.).

Contents:

1. General methods

2. Syntheses and Characterizations of Py-Phe-Phe-Lys-Lys-OH (K) and Py-Phe-Phe-Glu-Ala-Ala-OH (D)

3. Supporting Figures and Tables

1. General methods

Experimental Materials and Instruments. All materials were reagent grade or better. Annexin V-FITC Apoptosis Detection Kit were purchased from Beyotime Inc. (China). TNF-alpha, Interleukin (IL)-6, IL-8 ELISA kits were purchased from Multi Sciences Inc. (China). H&E staining kit, Masson's trichrome staining kit and Oil Red O staining kit were obtained from Beyotime Inc. (China). Dexamethasone were ordered from Macklin Biochemical Co. (Shanghai, China). All other starting materials were obtained from Sigma-Aldrich, Adamas, or Sangon Biotech. Commercially available reagents were used without further purification, unless noted. HPLC analyses were performed on a Shimadzu UFLC system equipped with two LC-20AP pumps and an SPD-20A UV/vis detector using a Shimadzu PRC-ODS column, or on an Agilent 1200 HPLC system equipped with a G1322A pump and an in-line diode array UV detector using an Agilent Zorbax 300SB-C18 RP column, with CH_3CN (0.1% of trifluoroacetic acid (TFA)) and ultrapure water (0.1% of TFA) as the eluent. Electrospray ionization (ESI) mass spectra were obtained on a Finnigan LCQ Advantage ion trap mass spectrometer (ThermoFisher Corporation). ^{1}H-NMR and ^{13}C-NMR spectra were recorded on a Bruker AV 400. Rheology test was carried on a Haake AR-G2 rheometer (TA Instruments), with angular frequency from 300.0 to 0.1000, 20 points per decade with 0.10000% strain for frequency sweep test, and with sweep from 0.010000 to 500, 20 points per decade with 6.283 rad/s angular frequency for strain sweep test. TEM images were recorded by transmission electron microscopy (TEM,

CM 200 electron microscope, Philips). Fluorescence spectra were obtained on a Hitachi FL-4600 fluorescence spectrophotometer (Hitachi High-Techonologies Corporation, Japan) with excitation wavelength set to 340 nm. Orbital fibroblasts (OFs) were obtained from primary cultures of orbital tissue from BALB/c mice and cell identification was performed. All animals received care according to the guidelines in the Guide for the Care and Use of Laboratory Animals of Nanjing Medical University (Nanjing, China). The procedures were approved by the Ethics Committee at the Institutional Animal Care and Use Committee of NJMU.

Rheology. After preparations of these four hydrogels (Gel K, Gel Dex/K, Gel D and Gel Dex/D), 1 mL of each hydrogel was taken and tested with a rheometer.

Transmission electron microscopy (TEM). Carbon-coated copper grids were glow-discharged to increase their hydrophilicity before use. After preparations of these four hydrogels (Gel K, Gel Dex/K, Gel D and Gel Dex/D), each of the hydrogel was diluted to 1 mM by PB (20 mM, pH 7.4) and the carbon-coated side of the grid was gently immersed in the solution for 1s. Then the grid was freeze-dried overnight and was then examined immediately.

Circular dichroism (CD). After preparations of these four hydrogels (Gel K, Gel Dex/K, Gel D and Gel Dex/D), each of the hydrogel was diluted to 250 μM by PB (20 mM, pH 7.4). Then they were examined by circular dichroism instrument immediately.

Critical aggregation concentration (CACs). Each of the hydrogelator was dispersed in PB (20 mM, pH 7.4) and then serial dilutions at concentrations ranging from 1.0 μM to 1000 μM were obtained. Plots of the fluorescence emission at 338 nm versus concentration revealed two regimes for each hydrogelator, indicating the CAC of 15.48 μM for Gel D, 15.84 for Gel Dex/D, and 16.20 μM for Gel K, and 34.06 μM for Gel Dex/K respectively.

Animals. Female BALB/c mice aged 8-10 weeks, weighing 20-35 g, were obtained from the Beijing Vital River Laboratory Animal Technology Co., Ltd.. The animal experiments were approved by the Institutional Animal Care and Use Committee of Nanjing Medical University with an affidavit of Approval of Animal Ethical and Welfare number of IACUC-2110024. All animals were kept in Animal Core Facility of Nanjing Medical University throughout the experiments (ACFNMU, Nanjing, Jiangsu, China). The animals were bred and housed in the pathogen-free animal facility and fed a standard normal diet and libitum with free access to water. The surgical specimens were handled according to the Declaration of Helsinki.

2. Syntheses and Characterizations of Py-Phe-Phe-Lys-Lys-OH (K) and Py-Phe-Phe-Glu-Ala-Ala-OH (D)

Scheme S1. The synthetic route for Py-Phe-Phe-Lys-Lys-OH (K).

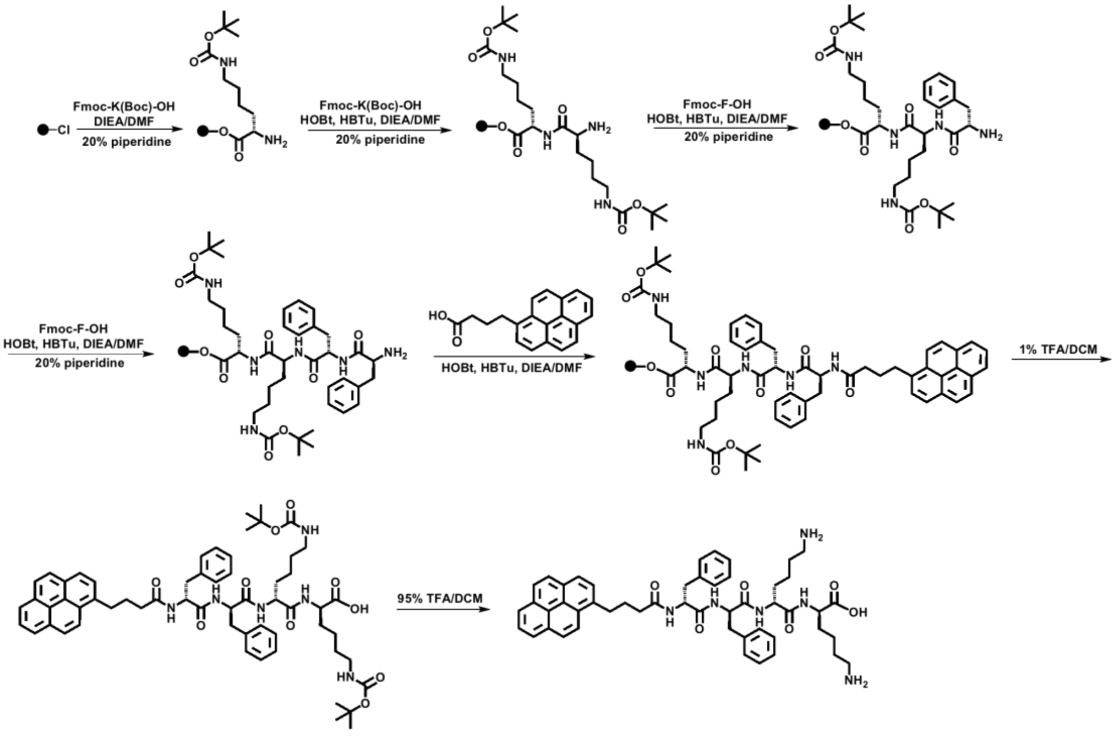

Solid phase peptide synthesis (SPPS) was used to prepare Py-Phe-Phe-Lys-Lys-OH (K), then its Boc groups were deprotected with dichloromethane (DCM, 300 μL) and triisopropylsilane (TIPS, 200 μL) in TFA (9.5 mL) for 3 h, and finally it was purified by HPLC using water-acetonitrile added with 0.1% TFA as the eluent. ^{1}H NMR (400 MHz, DMSO-d6) δ (ppm): 8.30-8.24 (m, 3 H), 8.20 (t, J = 9.0 Hz, 3 H), 8.14-8.11 (m, 3 H), 8.06 (d, J = 7.5 Hz, 2 H), 7.83 (d, J = 7.8 Hz, 1 H), 7.75 (s, 1 H), 7.19 (dt, J = 17.0, 7.7 Hz, 8 H), 7.08 (td, J = 7.0, 1.5 Hz, 2 H), 4.56 (dd, J = 8.3, 4.0 Hz, 2 H), 4.30 (dd, J = 13.4, 8.1 Hz, 1 H), 4.20-4.15 (m, 1 H), 3.12 (t, J = 7.7 Hz, 2 H), 3.00 (ddd, J = 33.8, 13.9, 3.9 Hz, 2 H), 2.83-2.68 (m, 6 H), 2.17 (t, J = 7.1 Hz, 2 H), 1.93-1.80 (m, 2 H), 1.71 (ddd, J = 21.0, 13.8, 5.3 Hz, 2 H), 1.60-1.49 (m, 6 H), 1.39-1.29 (m, 4 H) (Figure. S1). ^{13}C NMR (100 MHz,

DMSO-d_6) δ (ppm): 173.87 (1 C), 172.43 (1 C), 171.92 (2 C), 171.21 (1 C), 129.68 (9 C), 128.48 (9 C), 127.96 (2 C), 127.60 (1 C), 126.97 (1 C), 126.62 (1 C), 125.31 (2 C), 124.64 (2 C), 124.03 (1 C), 54.20 (2 C), 52.52 (1 C), 52.12 (1 C), 39.94 (1 C), 39.73 (1 C), 39.52 (1 C), 39.18 (1 C), 37.71 (1 C), 35.29 (1 C), 32.54 (1 C), 32.07 (1 C), 30.83 (1 C), 27.88 (1 C), 27.09 (1 C), 22.56 (2 C) (Figure. S2). MS: calculated for Py-Phe-Phe-Lys-Lys-OH (K) ($C_{50}H_{59}N_6O_6$) $[(M + H)]^+$: 839.4; obsvd. ESI-MS: m/z 839.4 (Figure. S3).

Scheme S2. The synthetic route for Py-Phe-Phe-Glu-Ala-Ala-OH (D).

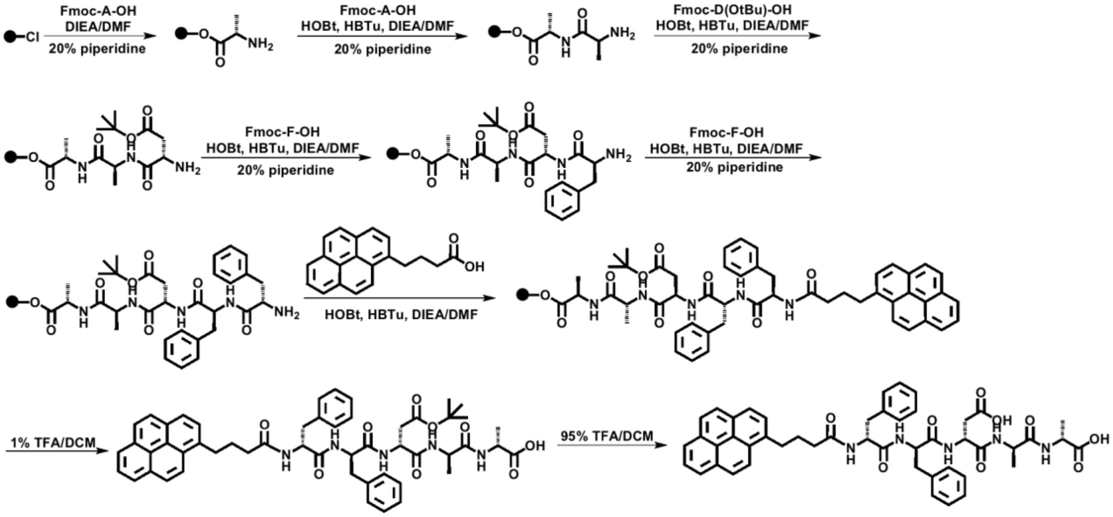

Solid phase peptide synthesis (SPPS) was used to prepare Py-Phe-Phe-Glu-Ala-Ala-OH (D), then its tBu group was deprotected with dichloromethane (DCM, 300 μL) and triisopropylsilane (TIPS, 200 μL) in TFA (9.5 mL) for 3 h, and finally it was purified by HPLC using water-acetonitrile added with 0.1% TFA as the eluent. ^{1}H NMR (400 MHz, DMSO-d6) δ (ppm): 8.38 (d, J = 7.6 Hz, 1 H), 8.28 (dd, J = 10.6, 5.2 Hz, 3 H), 8.20 (t, J = 8.3 Hz, 2 H), 8.16-8.10 (m, 4 H), 8.09-8.03 (m, 2 H), 7.84 (t, J = 6.8 Hz, 2 H), 7.27-7.14

(m, 8 H), 7.07 (t, J = 7.1 Hz, 2 H), 4.63-4.50 (m, 3 H), 4.32-4.26 (m, 1 H), 4.21-4.15 (m, 1 H), 3.12 (t, J = 7.8 Hz, 2H), 3.01 (ddd, J = 25.5, 13.9, 3.9 Hz, 2 H), 2.84-2.66 (m, 3 H), 2.56-2.52 (m, 1 H), 2.16 (t, J = 7.1 Hz, 2 H), 1.84 (tt, J = 13.7, 7.1 Hz, 2 H), 1.27 (d, J = 7.3 Hz, 3 H), 1.23-1.19 (m, 3 H) (Figure. S4). ^{13}C NMR (100 MHz, DMSO-d_6) δ (ppm): 174.42 (1 C), 172.26 (3 C), 171.92 (1 C), 171.56 (1 C), 170.35 (1 C), 138.55 (1 C), 138.05 (1 C), 137.07 (1 C), 131.35 (1 C), 130.90 (1 C), 129.69 (6 C), 128.48 (6 C), 127.96 (2 C), 127.60 (1 C), 126.95 (1 C), 126.64 (2 C), 125.31 (2 C), 124.65 (2 C), 124.05 (1 C), 54.16 (2 C), 49.92 (1 C), 48.38 (1 C), 47.93 (1 C), 37.89 (2 C), 36.40 (1 C), 35.30 (1 C), 32.55 (1 C), 27.88 (1 C), 18.65 (1 C), 17.53 (1 C) (Figure. S5). MS: calculated for Py-Phe-Phe-Glu-Ala-Ala-OH (D) ($C_{48}H_{50}N_5O_9$) $[(M + H)]^+$: 840.4; obsvd. ESI-MS: m/z 840.3 (Figure. S6).

3. Supporting Figures and Tables

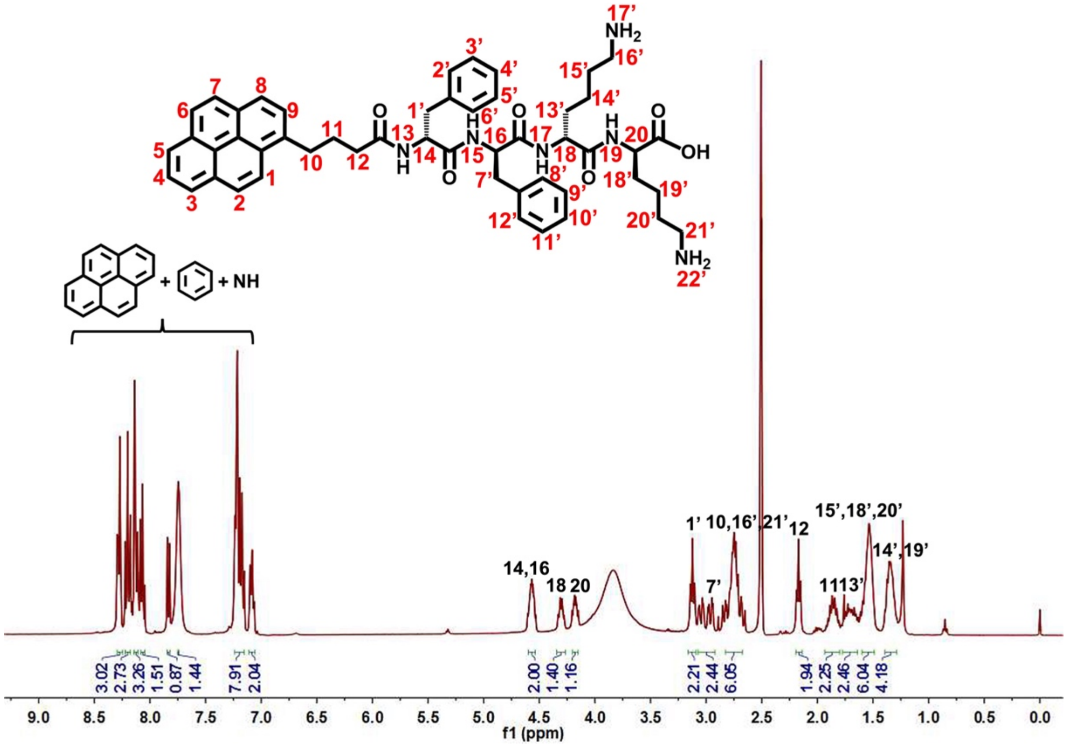

Figure S1. ^{1}H NMR spectrum of Py-Phe-Phe-Lys-Lys-OH (K) in DMSO-d_6.

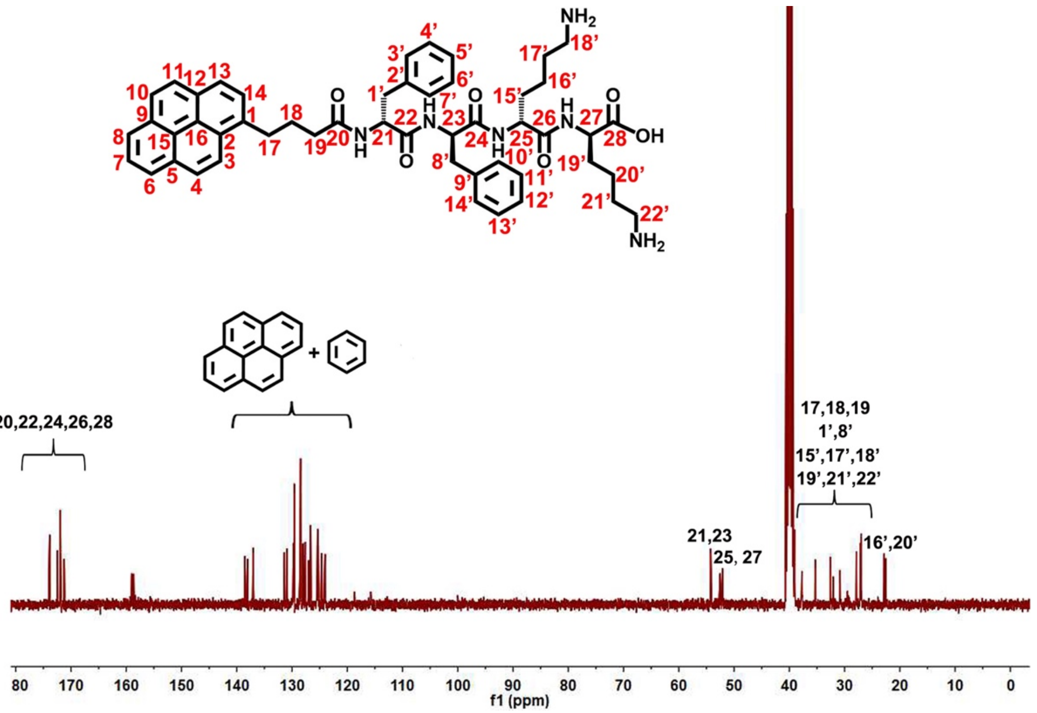

Figure S2. ^{13}C NMR spectrum of Py-Phe-Phe-Lys-Lys-OH (K) in DMSO-d_6.

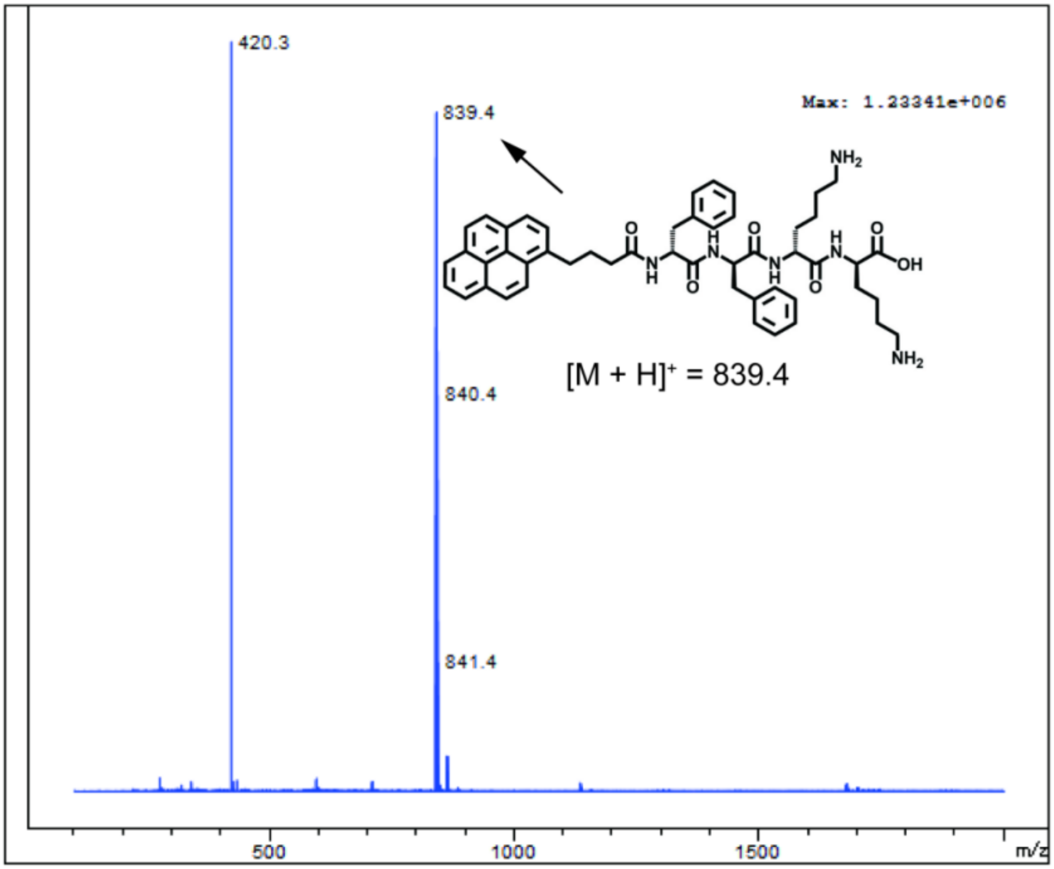

Figure S3. ESI-MS spectrum of Py-Phe-Phe-Lys-Lys-OH (K).

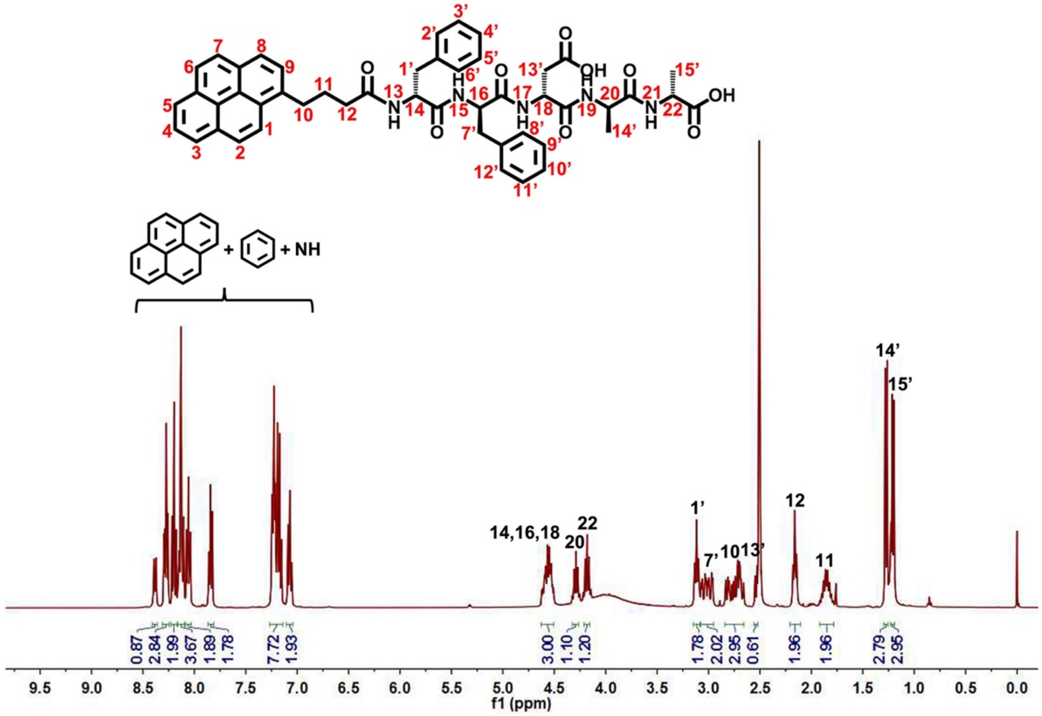

Figure S4. ^{1}H NMR spectrum of Py-Phe-Phe-Glu-Ala-Ala-OH (D) in DMSO-d_6.

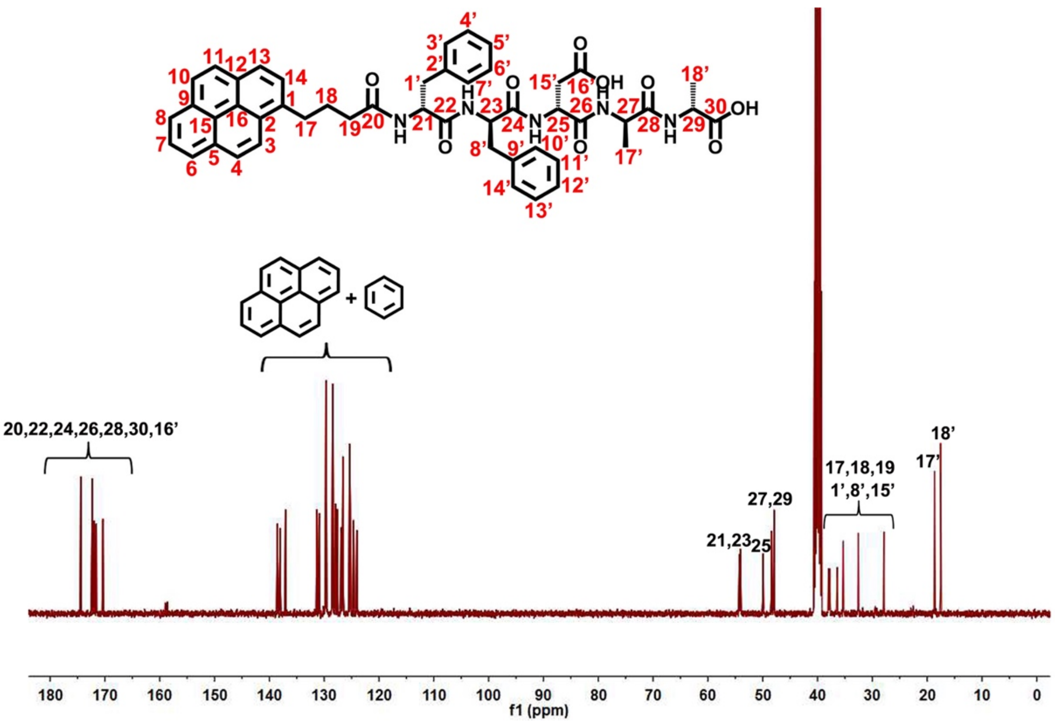

Figure S5. ^{13}C NMR spectrum of Py-Phe-Phe-Glu-Ala-Ala-OH (D) in DMSO-d_6.

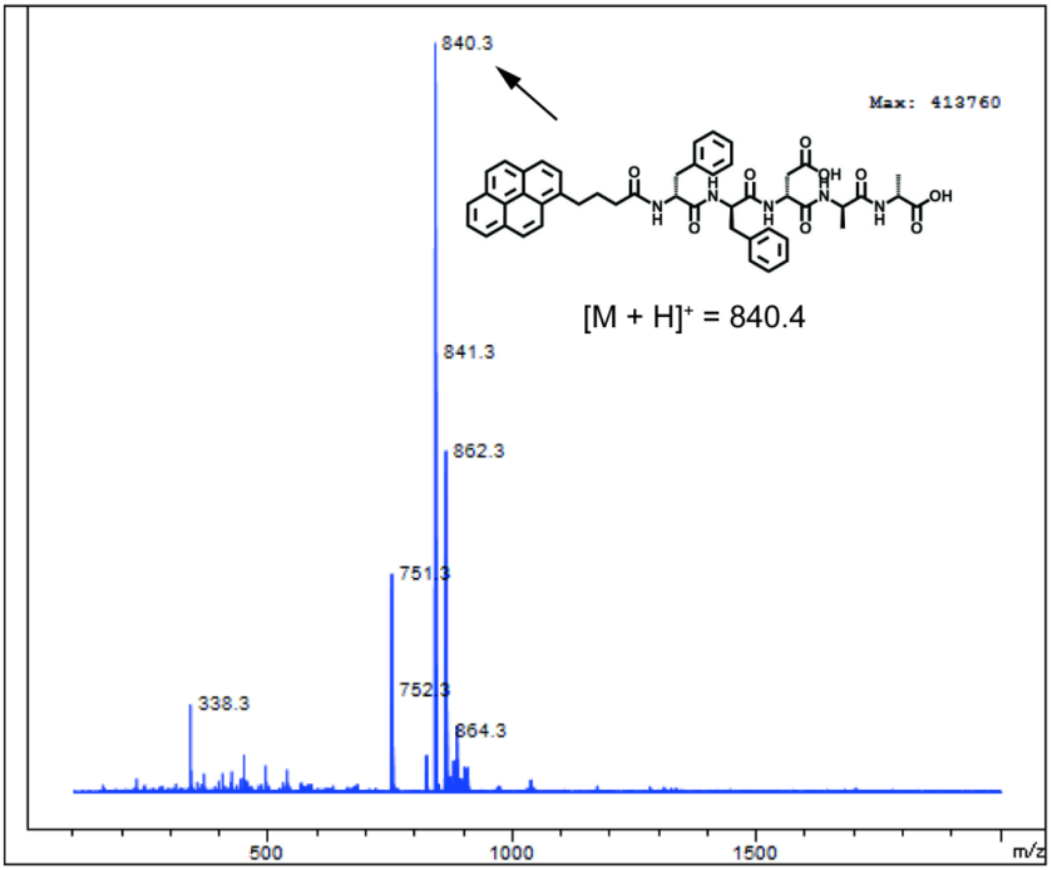

Figure S6. ESI-MS spectrum of Py-Phe-Phe-Glu-Ala-Ala-OH (D).

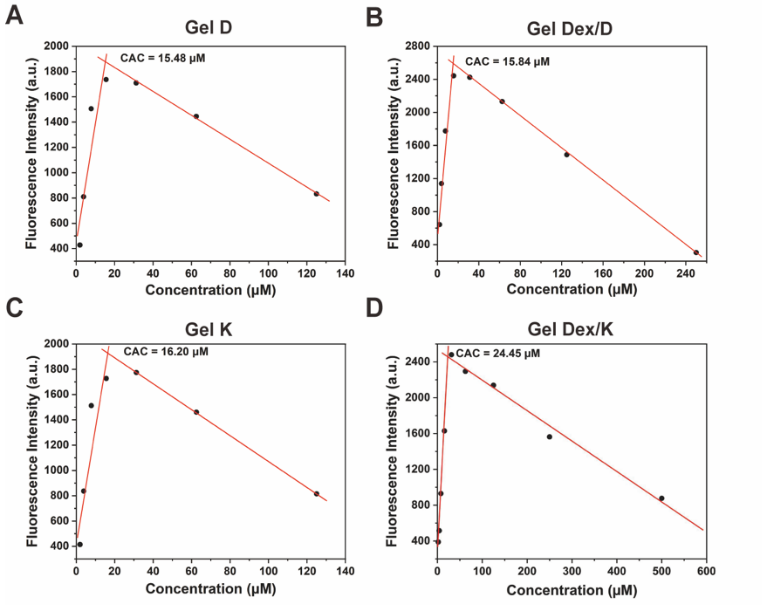

Figure S7. Concentration-dependent fluorescence intensity at 338 nm of dilutions of Gel D **(A)**, Gel Dex/D **(B)**, Gel K **(C)** and Gel Dex/K **(D)**.

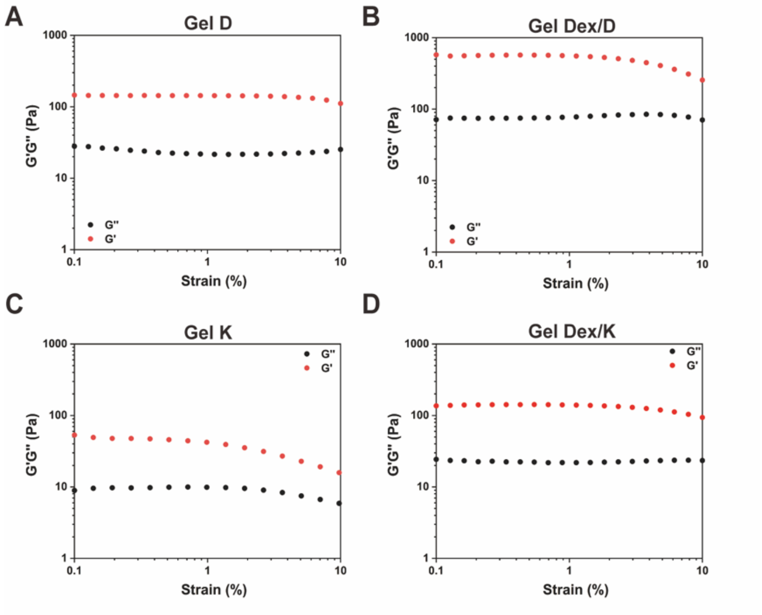

Figure S8. Characterizations of the dynamic strain scanning of Gel D, Gel Dex/D, Gel K and Gel Dex/K at 10mg/mL in PB (20 mM, pH 7.4), respectively. (25 ℃, frequency: 1 Hz). (A) Dynamic strain scanning of Gel D. **(B)** Dynamic strain scanning of Gel Dex/D. **(C)** Dynamic strain scanning of Gel K. **(D)** Dynamic stain scanning of Gel Dex/K.

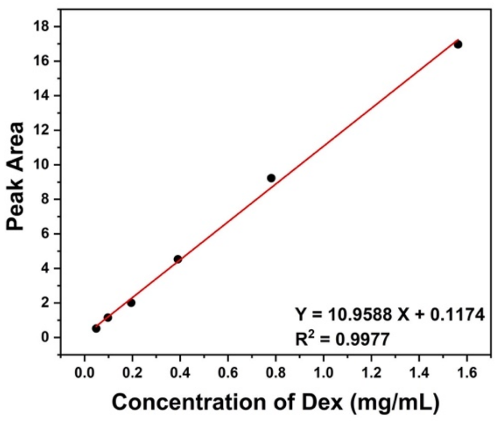

Figure S9. Plotting of the HPLC peak area vs. the concentration of Dex.

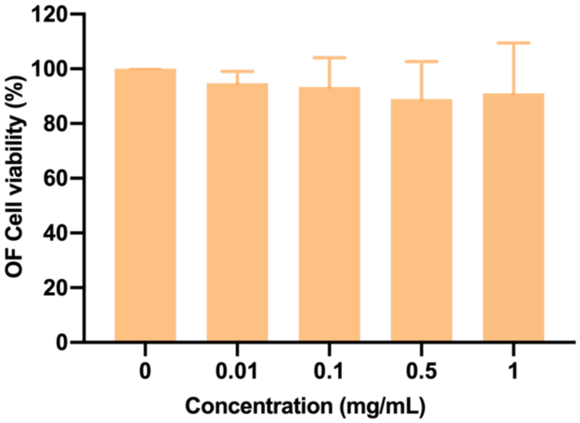

Figure S10. Cell viability of OF cells incubated with 0 - 1 mg/mL Dexamethasone for 24 hours. Data were expressed as mean ± SD. n = 5. *P < 0.05 vs Control.

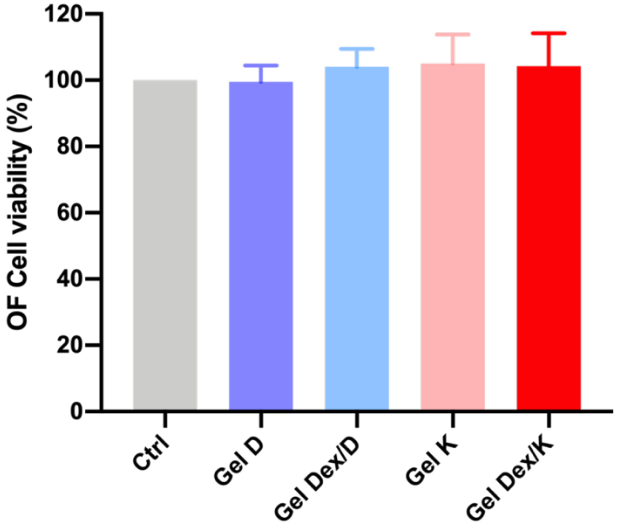

Figure S11. Cell viability of OF cells incubated with culture medium (Ctrl), 10mg/mL Gel D, 10mg/mL Gel Dex/D, 10mg/mL Gel K and 10mg/mL Gel Dex/K for 72 hours. Data were expressed as mean ± SD. n = 5. *P < 0.05 vs Control.

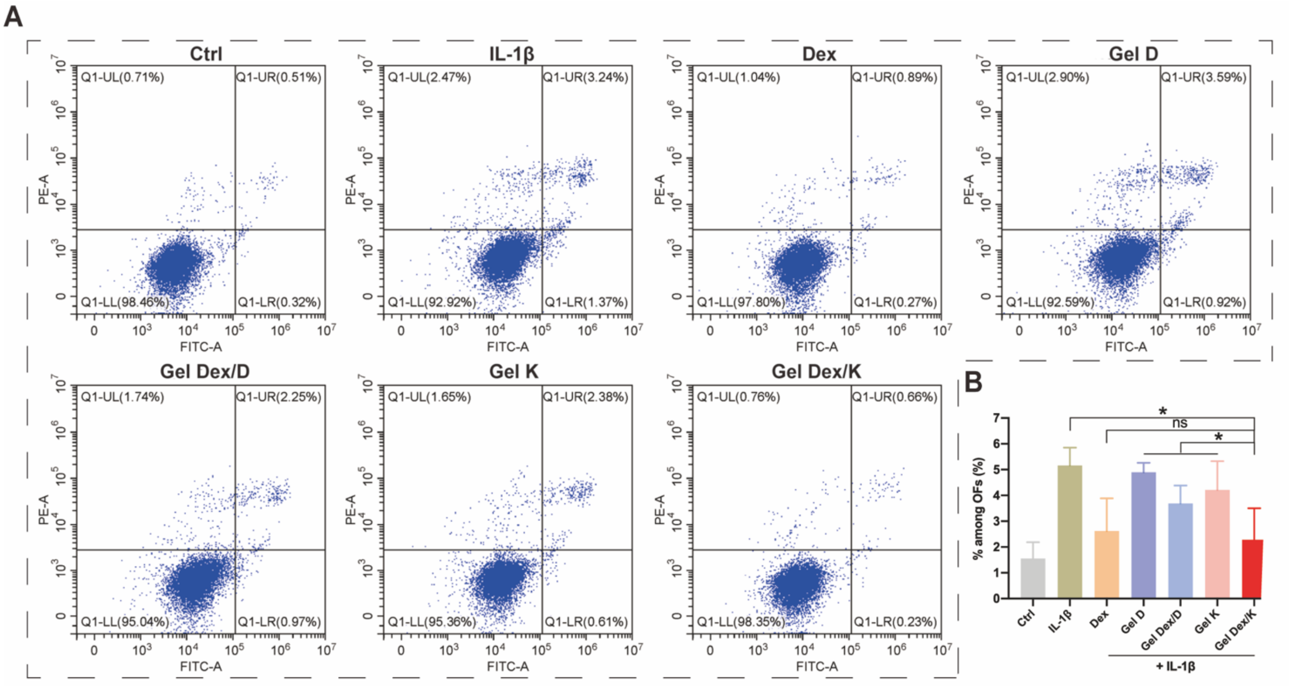

Figure S12. Apoptosis of OF cells in each group under simulated inflammatory environment *in vitro*. (A) Flow Cytometry Results. **(B)** Statistical analysis of apoptotic cells. Data were expressed as mean ± SD. n = 3. *P < 0.05 vs Control.

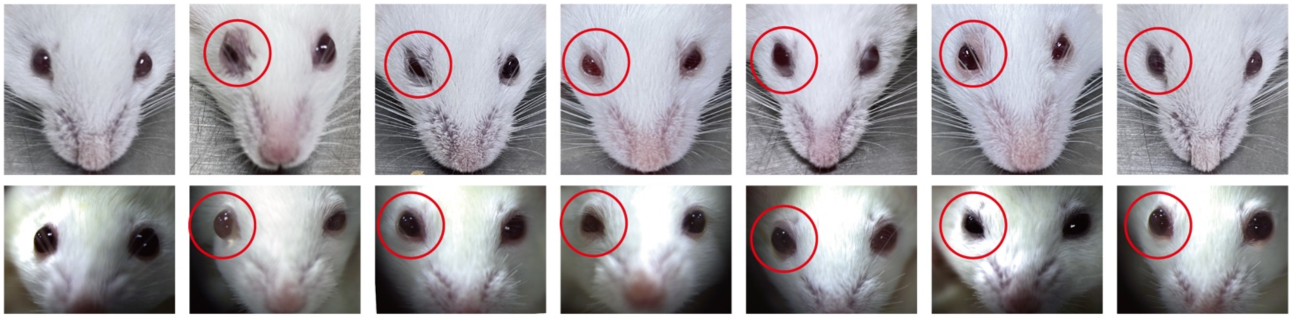

Figure S13. Bright-field photos (top row) and slit-lamp photos (second row) of the ocular surface of mice on the fifth day after treatment.

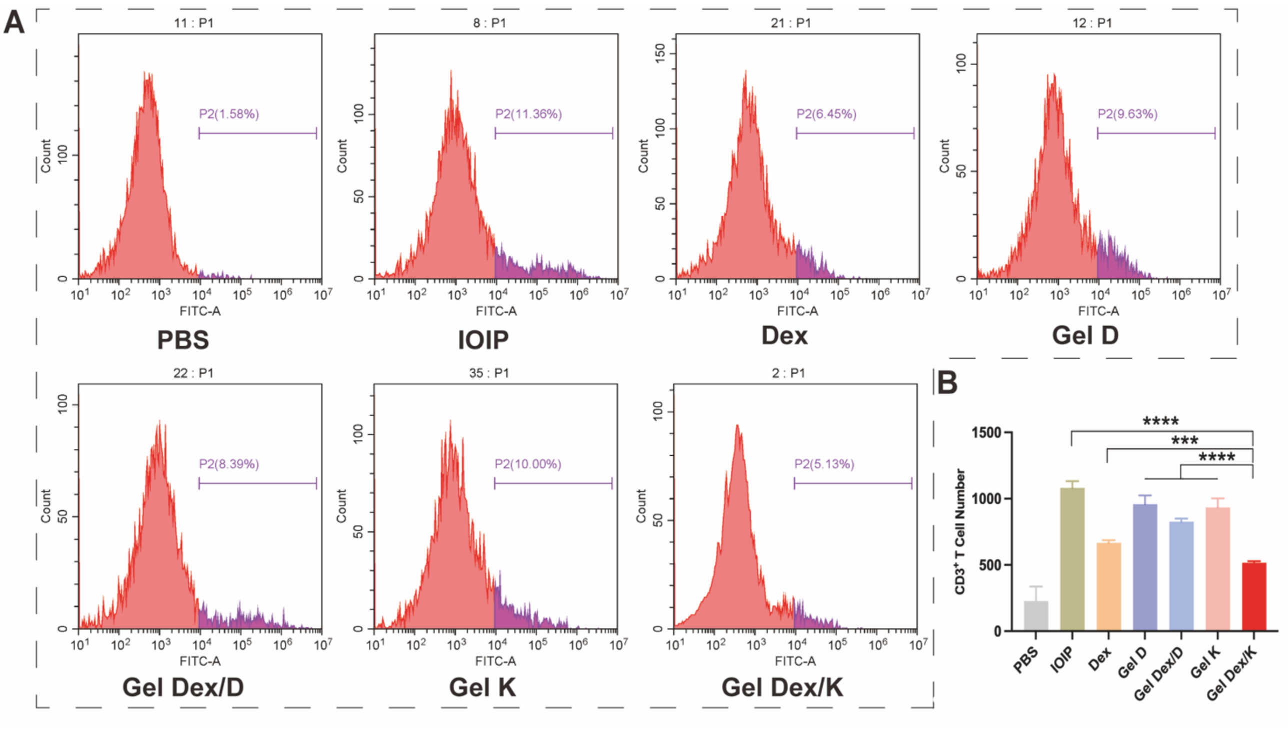

Figure S14. Flow cytometry results **(A)** and statistical analysis **(B)** of $CD3^+$ T cells in orbital tissues of mice in an *in vitro* simulated inflammatory environment. Data were expressed as mean ± SD. n=3. $^{*}P < 0.05$, $^{**}P < 0.01$, $^{***}P < 0.001$, $^{****}P < 0.0001$.

Table S1. HPLC condition for the purification of Py-Phe-Phe-Glu-Ala-Ala-OH (D).

Time (min)	Flow (mL/min)	H_2O% (0.1% TFA)	CH_3CN% (0.1% TFA)
0	12.0	50	50
3	12.0	50	50
35	12.0	5	95
37	12.0	5	95
38	12.0	50	50
40	12.0	50	50

Table S2. HPLC condition for the purification of Py-Phe-Phe-Lys-Lys-OH (K).

Time (min)	Flow (mL/min)	H_2O% (0.1% TFA)	CH_3CN% (0.1% TFA)
0	12.0	60	40
3	12.0	60	40
35	12.0	5	95
37	12.0	5	95
38	12.0	60	40
40	12.0	60	40

Table S3. HPLC condition for Figure. S9.

Time (min)	Flow (mL/min)	H_2O% (0.1% TFA)	CH_3CN% (0.1% TFA)
0	3.0	70	30
3	3.0	70	30
35	3.0	10	90
37	3.0	10	90
38	3.0	70	30
40	3.0	70	30
